# Patellar Tendon Rupture 12 Years after ACL Reconstruction with BPTB Autograft

**DOI:** 10.1155/2023/5591956

**Published:** 2023-09-27

**Authors:** Christopher C. Paiz, Dustin L. Richter

**Affiliations:** The University of New Mexico Hospital, Department of Orthopaedics, Albuquerque, New Mexico, USA

## Abstract

We present a case of a 33-year-old male with a history of anterior cruciate ligament reconstruction (ACLR) with bone-patellar tendon-bone (BPTB) autograft and prior ipsilateral hamstring harvest, who presented with a complete patella tendon rupture (PTR) 12 years after ACLR. The patient underwent a successful patellar tendon (PT) repair augmented with Achilles tendon allograft and cerclage with nonabsorbable suture tape. PTR after ACLR with BPTB autograft is rare, particularly in patients over a decade out from the index procedure, but can occur. This case report highlights a novel technique for PT repair following BTB ACLR in a hamstring deficient knee.

## 1. Introduction

Patellar tendon rupture (PTR) is a rare complication following anterior cruciate ligament reconstruction (ACLR) with ipsilateral bone-patellar tendon-bone (BPTB) autograft. The most commonly reported mechanism of injury is falling onto a flexed knee [1–4], although jumping and twisting mechanisms of injury have also been described [5, 6]. The reported incidence of PTR following ACLR with ipsilateral BPTB autograft ranges from 0.05% to 0.24% [1, 5, 7]. The majority of tears occur in the early postoperative period, but there have been six reports of tears occurring in the 7-month to 10-year range since this complication was first described by Bonamo et al. in 1984 [2–6, 8–10]. To our knowledge, this is the first report of a PTR greater than 10 years after ACLR with BPTB autograft in a knee with prior hamstring tendon (HT) harvest for an elbow ulnar collateral ligament reconstruction.

The patient was informed that data related to the case would be submitted for publication, and he provided consent. This study was deemed exempt by the local institutional review board.

## 2. Case Report

The patient is a 33-year-old male who presented to clinic 5 days after an injury sustained to the right knee while playing basketball. The patient reported that he planted the foot and went to turn and his knee gave way. He had immediate pain, swelling, and inability to perform a straight leg raise. His surgical history includes a right elbow ulnar collateral ligament (UCL) reconstruction with right knee hamstring autograft at age 19 and a right knee ACLR with ipsilateral BPTB autograft at age 21. He is a nonsmoker, has no chronic medical conditions, and works as an engineer. He played division II baseball in college and continues to lead a very active lifestyle.

**Figure 1:**
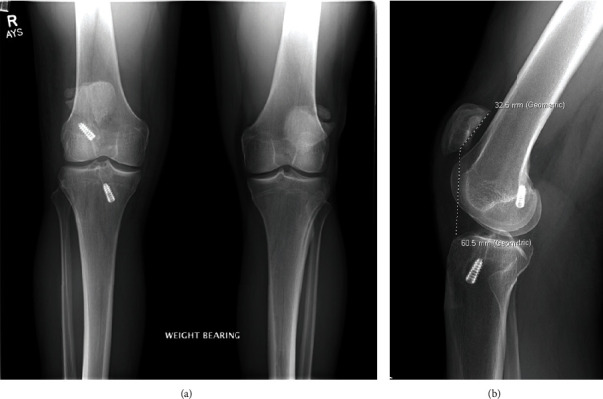
(a) Preoperative AP view of bilateral knees demonstrating bilateral bipartite patella and right knee ACL reconstruction. (b) Preoperative lateral view of the right knee demonstrating patella alta with a Caton-Deschamps ratio of 1.86 in the setting of prior ACL reconstruction.

On physical exam, he had a high riding patella, a palpable gap at the inferior pole of the patella, and moderate effusion. He was unable to perform a straight leg raise. He was otherwise ligamentously stable. Anterior-posterior (AP) and lateral radiograph showed patella alta with a Caton-Deschamps ratio of 1.86 and a bipartite patella bilaterally (Figure 1).

A diagnosis of patella tendon rupture was made, and the patient was taken to the operating room 48 hours later. The patient had a negative Lachman test on exam under anesthesia. The previous patellar incision was used and extended. The patellar tendon (PT) was completely torn predominately from the inferior pole of the patella. Additionally, severe disruption of the medial and lateral retinacular tissue and the central defect in the PT was observed.

**Figure 2:**
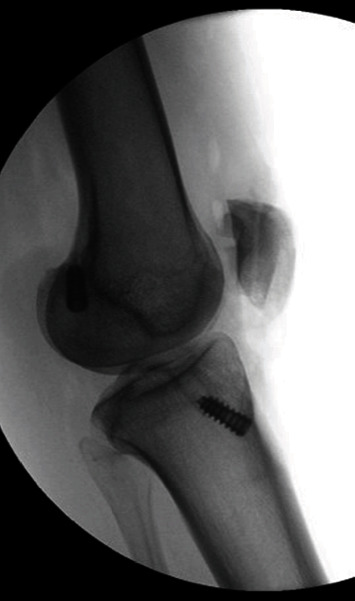
Intraoperative fluoroscopic image demonstrating correction of the patella alta.

The edges of both the distal and proximal PT stumps were debrided. The patella was mobilized distally, and the Caton-Deschamps ratio was checked with intraoperative fluoroscopy (Figure 2) [11]. The primary repair was performed with a #2 nonabsorbable suture in a Krakow configuration on the medial, central, and lateral aspects. Three longitudinal transosseous tunnels were drilled from distal to proximal through the patella, and sutures were passed through these tunnels. The patient was brought to full extension, and square knots were tied over the bone bridge proximally. The patient was flexed intraoperatively up to about 45 degrees of flexion without any gapping noted at the inferior patella. The retinacular tears on both the medial and lateral aspects of the knee were repaired.

**Figure 3:**
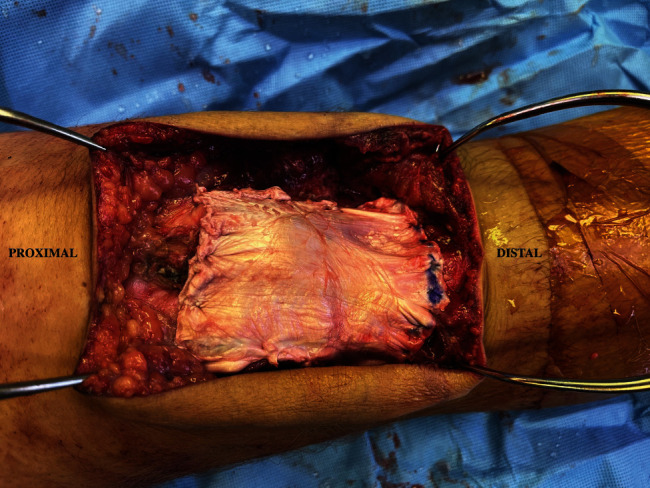
Intraoperative image of soft tissue Achilles allograft used to reinforce patellar tendon repair.

Given the patient's young age, activity level, and tension placed on the primary repair, backup fixation with a nonabsorbable suture tape cerclage was used. The suture tape was passed through a transosseous tunnel in the tibial tubercle and secured over the superior pole of the patella. Biologic and mechanical augmentation with Achilles tendon allograft was used to improve healing and support the PT repair in the setting of central tendon harvest site pathology from the ACLR (Figure 3). The soft tissue portion of an Achilles allograft was used and soaked in a vancomycin-wrapped sponge prior to use. The allograft was placed over the patella and PT and sutured to the surrounding tissue while under tension. The patient was placed in a knee immobilizer for the first month, and then, gradual knee flexion was achieved over the next several months with avoiding hyperflexion until the 3-month mark.

The patient remained pain free throughout subsequent follow-up visits. At the final 10-month follow-up, the patient rated pain 0/10, he was functioning at 90% of preinjury activity level, and overall satisfaction with his knee was 7/10 on a visual analog scale (VAS). He admitted that he had mostly stopped doing prescribed strength and flexibility exercise at around 4 months. On exam, ROM was from 1° short of full extension to 135° of flexion. Strength was 5/5 on resisted knee extension. Ligamentous exam was normal. Mild atrophy was observed in the right thigh. No patellar instability or joint effusion was present. The International Knee Documentation Committee (IKDC) Objective Knee Examination Form was used for the final assessment, and his overall score was “normal.” He scored “severely abnormal” on the functional test because his one leg hop distance in his right leg was less than 50% of his left leg.

The patient's Lysholm Knee Score was 95; the 5-point deduction was due to swelling of the knee after vigorous activity and slight problems in squatting. The Tegner Activity Scale (TAS) decreased from 7 preinjury to 4 postinjury. The IKDC subjective knee score was 65.5. He scored a 91.7% on the ACL-Return to Sport after Injury (ACL-RSI) Scale and noted some frustration over having to consider his knee with respect to sport.

**Figure 4:**
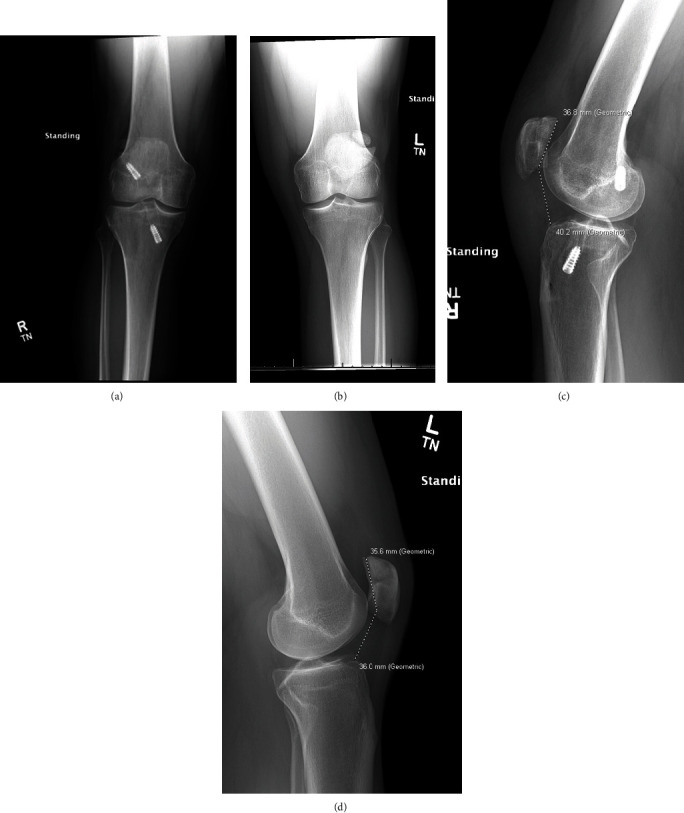
(a, b) Postoperative AP radiograph at 10 months of the right (operative) knee and contralateral left knee for comparison. (c, d) Postoperative lateral radiograph at 10 months of the right (operative) knee with a C-D ratio of 1.1 compared to the contralateral left knee with a C-D ratio of 1.0.

AP and lateral radiographs were obtained of bilateral knees at final follow-up (Figure 4). Mild arthritic changes were observed bilaterally, but no other significant abnormalities were observed. The Caton-Deschamps ratio was 1.1 on the right and 1.0 on the contralateral left.

## 3. Discussion

PTR after ACLR with BPTB autograft is rare, most often occurring in the early postoperative period, and is typically associated with falling onto a flexed knee; however, there are several reported cases of jumping and twisting mechanisms of injury and ruptures occurring up to a decade out from surgery [1, 6, 9]. After PT harvest, a defect exists in the central one-third of the tendon, predisposing the knee to a “Z”-shaped tear pattern where the proximal end is torn medially and the distal segment is torn laterally [8]. The central defect, tear patterns, and quality of the remaining tissue after BPTB autograft create a unique repair challenge that is not well addressed in native PTR literature [6, 12].

PT reconstruction with ipsilateral hamstring autograft is the preferred surgical technique in chronic PTR [13, 14]. The most frequently described repair technique involves freeing the hamstrings proximally and leaving the distal tendon intact at the pes anserinus, creating a strong anchor. Two transosseous tunnels are drilled medial to lateral, distally at the tibia just deep to the tibial tubercle and proximally at the midpoint of the patella. The tendon is passed through the tunnel at the patella and brought distally to the lateral tibial tunnel and secured [13–15]. In a systematic review of six studies utilizing this technique, five studies reported range of motion (ROM) with a mean of 128°, three studies reported a postoperative Lysholm with a mean of 90.6, and two studies reported IKDC scores with a mean improvement of 50.5 [13]. Reconstruction with ipsilateral HT is an excellent technique, but nonviable for a patient with prior hamstring harvest.

The techniques described in the reported cases of late PTR after ACLR with BPTB include primary repair alone, augmentation with nonabsorbable suture tape, BPTB allograft and wire loops, HT augmentation, and 2 cases of augmentation with wire cerclage [3–6, 9]. Miroslav et al. elected to use a BPTB allograft and wire loop technique to repair a PTR occurring 7 months after ACLR with BPTB [5]. Of the reported cases, this technique is most similar to the technique described by the authors. Their patient's knee flexion progressed further by 3 months, but at final follow-up, knee ROM, quadriceps strength, extensor lag, and patellar height were near identical. However, the technique described by Miroslav et al. and all techniques with wire fixation have the significant drawback of requiring an additional surgery to remove broken screws and wires [5].

Lissy and Patel elected to use nonabsorbable suture tape augmentation in their PT repair, although with a different technique than the authors and in their case a portion of the patella tendon remained attached to the inferior pole of the patella [3]. The tendon repair was reinforced by suture tape in an “X” configuration with anchors medial and lateral to the tendon at the inferior pole of the patella and at the tibial tubercle [3]. Their patient had excellent functional outcomes and ROM at 6 months, although it should be noted the patient was a decade younger than the patient described by the authors.

We report the first known case of PTR 12 years after ACLR with BPTB autograft in a hamstring deficient knee. This report demonstrates that although PTR after ACLR with BPTB is rare, it can occur and that the complexity of the repair is increased in patients with coexisting tendon harvest pathology about the knee. The authors elected to use Achilles allograft augmentation given the broad nature of the tendon in covering the repair site and potential for biologic incorporation, although other allograft or mesh options could have been utilized for the reconstruction. In addition, many modern rehabilitation protocols promote early range of motion following primary tendon repair in healthy tissue; however, given the prior BPTB harvest defect and overall tenuous quality of the native tissue, we delayed the initiation of knee flexion to encourage early healing of the extensor mechanism in extension. A paucity of literature exists describing late PT repair after ACLR with BPTB, and to our knowledge, there are no reported examples or repair in patients with prior hamstring harvest. This case highlights an augmentation strategy that may be utilized with excellent postoperative outcomes.
